# Matching of advanced undergraduate medical students’ competence profiles with the required competence profiles of their specialty of choice for postgraduate training

**DOI:** 10.1186/s12909-023-04632-3

**Published:** 2023-09-07

**Authors:** Lea Jebram, Sarah Prediger, Viktor Oubaid, Sigrid Harendza

**Affiliations:** 1 III. Department of Internal Medicine, University Medical Centre Hamburg-Eppendorf, Hamburg, Germany; 2 German Aerospace Centre (DLR), Hamburg, Germany

**Keywords:** Competence, Medical specialty, Medical student, Residency, Postgraduate medical education, Specialty selection, Undergraduate medical education

## Abstract

**Background:**

Matching between undergraduate students and their chosen specialty has implications for their personal job satisfaction and performance as well as societies’ needs regarding health care quality. Knowledge regarding student-specialty fit can help improve students’ decisions and detect potential deficiencies in specific competences. In this study, we compare self-assessed competence profiles of medical students close to graduation with the competence profiles of their specialty of choice for postgraduate training.

**Methods:**

Self-assessed competence profiles were collected with the modified requirement-tracking (R-Track) questionnaire from 197 final-year medical students close to graduation in 2022. To determine student-specialty fit, difference scores between students’ self-assessed competences and physicians’ requirements for specific specialties were calculated across the R-Track’s six competence areas “Motivation”, “Personality traits”, “Social interactive competences”, “Mental abilities”, “Psychomotor & multitasking abilities”, and “Sensory abilities”, which were assessed on a 5-point Likert scale (1: “very low” to 5: “very high”). Mean difference scores across competence areas were calculated and compared between specialties with multivariate analysis of variance. Student-specialty fit was also calculated independent of students’ choices.

**Results:**

The competence area “Motivation” scored highest for both students and physicians across specialties. However, students’ scores were lower than physicians’ requirements for “Motivation” as well as “Personality traits” across all specialties. Difference scores for “Social interactive competences” were either close to zero or showed higher scores for students. A similar competence pattern for internal medicine, general medicine, paediatrics, and gynaecology was identified with higher than required student scores for “Mental abilities”, “Psychomotor & multitasking abilities”, and “Sensory abilities”. All other specialties showed higher physicians’ requirements for at least one of these competence areas. Independent of students’ specialty choice, we found the highest difference score in favour of student scores for general medicine (0.31) and the lowest difference score for internal medicine (-0.02).

**Conclusions:**

Students’ competence profiles overall show better fit with person-oriented specialties. “Mental abilities”, “Psychomotor & multitasking abilities”, and “Sensory abilities” show higher requirement scores for more technique-oriented specialties. Students interested in such specialties could focus more on basic skill development in undergraduate training or will develop specific skills during residency.

**Supplementary Information:**

The online version contains supplementary material available at 10.1186/s12909-023-04632-3.

## Background

The choice of a medical specialty represents one of the most important decisions during medical education [1]. Only few of those choices are changed once made [2, 3]. This importance aggravates considering that person-job fit in the medical profession does not only impact personal wellbeing, job satisfaction, and performance. It can consequently affect the overall healthcare system due to lower quality care, physician burn out, or turnover, if unfitting choices were made [4]. Thus, ideally, medical students should choose a specialty they are optimally qualified and motivated for. Factors considered by medical students in order to make their decision for a specialty for residency training are numerous and include aspects like interest [5], exposure to a certain specialty [6], amount of patient contact [7], work-life balance [8], income [9], or prestige [10]. Identifying these factors and understanding how students make their decisions can help guide them to make a good choice towards person-job fit. Furthermore, to recruit enough skilled residents is especially important for specialties lacking applications, e.g., general medicine and paediatrics [11, 12].

Specialties attracting sufficient applications usually opt for accepting students with higher grades in relevant areas or other performance-based criteria like status of medical school [13] or further qualifications [14]. Thus far, factors relevant for specialty choice were centred around what specialties have to offer and if that fits the students’ needs and preferences. There are, however, also factors attributing to the requirements of a specialty and students’ aptitudes regarding competences [7, 15]. Other aspects like personality [16], in certain specialties being associated with specific personality traits [17], personality types [18], or personality characteristics like empathy [19] are also being taken into account. Choosing the specialty that students fit in best though hinges on the assumptions students have about specialties which can deviate from real circumstances regarding the requirements and decisive aspects of a specialty [20]. Exposure to a specialty can improve understanding of the specialty’s requirements or work conditions [21] and potentially foster interest [22]. However, it will still be subjective and therefore does not suffice for the purpose of matching requirements and students’ characteristics efficiently. In order to also be able to test the student-specialty fit and use compatibility as a tool to improve the decision making towards better fit, students’ self-assessments can be compared with specialty requirement profiles. Such profiles have already been defined for anaesthesiology [23], nephrology [24] and other medical specialties [25].

Competence profiles of specialties demonstrate the differences between specialties and make it possible to match students to their targeted specialty to determine which offers the best fit. On an individual level this can help students decide which specialty they want to choose, while on a professional level it facilitates distributing students according to their competences thus improving job satisfaction [26]. Fitting students and specialties could also highlight deficiencies in undergraduate medical training when specialties show a low matching rate also in comparison to others. Similar thoughts have already been investigated for dermatology [27], ophthalmology [28] or psychiatry [29]. This study aims to use specialty competence profiles and students’ self-assessment of competences to evaluate students’ fit with their chosen specialty across a variety of specialties. We further investigate differences between specialties regarding the fit of students’ competences to the requirements of the specialties, thereby potentially demonstrating educational needs prior to specialty selection in order to improve student-specialty fit.

## Methods

### Study design and participants

Between September and December 2022, final-year medical students of the region of Northern Germany who had participated in an information event on how to apply for residency were given the possibility to participate in a digital survey of self-assessed competence profiles. Additionally, sociodemographic data (age and gender) were collected and participants named their first and second choice of specialty for residency training. Participation was voluntary and anonymous, and all participants provided informed written consent for participation in this study which was approved by the Ethics Committee of the Chamber of Physicians, Hamburg (PV3649). For data analysis, the competence profiles of the participating students were compared with the competence profiles physicians had provided for their respective specialty in a previous study [25].

### Instrument

The Requirement-Tracking questionnaire (R-Track) was used for medical students’ self-assessment of competences. Originally designed for assessment of airline pilots’ competences [30], the questionnaire was previously adapted for health care professionals [25] and also for health care professionals’ self-assessment [31]. Based on established instruments like the Fleishman Job Analysis Survey [32], the R-Track questionnaire aims to assess a broader set of necessary skills and abilities required to successfully fulfill professional tasks. Using a 5-point Likert scale (1: “very low” to 5: “very high”) the R-Track assesses 63 facets of competence, i.e., individual abilities, skills, personality traits, and motivational aspects relevant for successful performance [33], assigned to six areas of competence (“Motivation”, “Personality traits”, “Social interactive competences”, “Mental abilities”, “Psychomotor & multitasking abilities”, “Sensory abilities”). R-Track items per competence area can be obtained from Additional file 1. For the comparison of competence profiles defined by physicians existing data from a previous study [25] were used. The R-Track items from the expert questionnaire were also assessed on a 5-point Likert scale (1: “very low importance” to 5: “very high importance”). Internal consistency (Cronbach’s α = 0.88) was comparable with previous R-Track assessments.

### Data analysis

Data were processed using R Version 4.2.2. For analysis regarding different specialties, we included only specialties with sufficient data (i.e., at least *n* = 7 participants’ first choice of a specialty, following Kleinmann et al.’s suggestion for job analysis instruments [34]) and available data from the expert study for a respective specialty [25]. Mean scores and standard deviations for all competence areas were computed only for specialties with sufficient data as well as average scores across all areas and specialties. Competence area mean scores of students and physicians were compared according to the students’ respective specialty choice and difference scores obtained. Difference scores were computed subtracting physician from student scores, thus resulting in negative scores when expectations from physicians are higher and positive scores when student self-assessment is higher. Additionally, difference scores were computed across all students independent of their specialty choice to determine best fit overall. To rule out possible demographic factors influencing difference scores, we ran a multivariate multiple regression model with interaction of independent variables for all six competence areas. Across all eligible specialties difference scores were compared for each of the six competence areas using multivariate analysis of variance (MANOVA). To further determine where difference scores deviate between specialties, we used univariate analysis of variance (ANOVA) and applied Bonferroni correction to account for multiple testing. For post-hoc comparisons between specialties we computed Tukey HSD tests. The general α-level was set at 0.05.

## Results

Overall, 197 final-year medical students (age = 27.5 ± 4.0 years, male = 30%, female = 70%) participated. Students named 17 different specialties as their individual specialty of choice for residency training. Specialties with n < 7 participants that could not be used for analysis were intensive care medicine, neurology, neurosurgery, occupational medicine, ophthalmology, otolaryngology, psychiatry, and urology. Sufficient data was available for *n* = 9 specialties which were included in the analysis: internal medicine (*n* = 44), general medicine (*n* = 24), paediatrics (*n* = 21), anaesthesiology (*n* = 18), gynaecology (*n* = 17), surgery (*n* = 14), orthopaedics (*n* = 8), dermatology (*n* = 7), and radiology (*n* = 7), resulting in a total of 160 participants (age = 27.8 ± 4.2 years, male = 28%, female = 72%). Highest and lowest mean scores across all specialties in both groups (student and expert physician) were obtained for competence areas “Motivation” (*M*_*students*_ = 3.91 ± 0.54, *M*_*physicians*_ = 4.36 ± 0.16) and “Sensory abilities” (*M*_*students*_ = 3.61 ± 0.58, *M*_*physicians*_ = 3.51 ± 0.22), respectively. Multiple regression showed no significant effect of sex or age on difference scores for all competence areas but “Personality traits” (b = 0.029, *p* = 0.005), where difference scores become greater with increasing age, indicating better personality fit of students. Full display of means for both groups for the included specialties and difference scores between students and physicians can be obtained from Table 1.

**Table 1:** Means and difference scores across competence areas and specialties

	**Competence areas**									
**Specialties**	**Motivation**			**Personality traits**			**Social interactive competences**			
	Students M ± SD	Physicians M ± SD	Diff	Students M ± SD	Physicians M ± SD	Diff	Students M ± SD	Physicians M ± SD		Diff
**Internal medicine** ***n***_***s***_ = 44 ***n***_***p***_ = 20	3.93 ± 0.51	4.36 ± 0.39	-0.43	3.65 ± 0.33	3.89 ± 0.42	-0.24	3.76 ± 0.31	3.91 ± 0.41		-0.15
**General medicine** ***n***_***s***_ = 24 ***n***_***p***_ = 11	3.82 ± 0.66	3.93 ± 0.45	-0.11	3.74 ± 0.33	3.85 ± 0.49	-0.11	3.70 ± 0.34	3.66 ± 0.32		0.04
**Paediatrics** ***n***_***s***_ = 21 ***n***_***p***_ = 7	4.02 ± 0.40	4.20 ± 0.58	-0.18	3.89 ± 0.35	3.92 ± 0.54	-0.03	3.78 ± 0.33	3.79 ± 0.38		-0.01
**Anaesthesiology** ***n***_***s***_ = 18 ***n***_***p***_ = 11	3.93 ± 0.56	4.38 ± 0.43	-0.45	3.81 ± 0.35	4.01 ± 0.44	-0.20	3.86 ± 0.28	3.79 ± 0.60		0.07
**Gynaecology** ***n***_***s***_ = 17 ***n***_***p***_ = 7	3.73 ± 0.35	4.06 ± 0.41	-0.33	3.61 ± 0.36	3.75 ± 0.38	-0.14	3.67 ± 0.35	3.67 ± 0.36		0.00
**Surgery** ***n***_***s***_ = 15 ***n***_***p***_ = 14	3.94 ± 0.75	4.54 ± 0.44	-0.60	3.95 ± 0.22	4.14 ± 0.47	-0.19	3.85 ± 0.39	3.85 ± 0.50		0.00
**Orthopaedics** ***n***_***s***_ = 8 ***n***_***p***_ = 11	3.83 ± 0.69	4.55 ± 0.37	-0.72	3.58 ± 0.55	4.00 ± 0.45	-0.42	3.93 ± 0.49	3.83 ± 0.39		0.10
**Dermatology** ***n***_***s***_ = 7 ***n***_***p***_ = 10	4.20 ± 0.53	4.52 ± 0.53	-0.32	3.76 ± 0.29	3.92 ± 0.51	-0.16	3.84 ± 0.26	3.91 ± 0.36		-0.07
**Radiology** ***n***_***s***_ = 7 ***n***_***p***_ = 9	3.89 ± 0.41	4.73 ± 0.28	-0.84	3.70 ± 0.47	4.17 ± 0.51	-0.47	3.79 ± 0.57	4.22 ± 0.59		-0.43
**Mean**	3.90	4.36	-0.44	3.73	3.96	-0.22	3.78	3.85		-0.04
	**Competence areas**									
**Specialties**	**Mental abilities**			**Psychomotor & multitasking abilities**			**Sensory abilities**			**Overall**
	Students M ± SD	Physicians M ± SD	Diff	Students M ± SD	Physicians M ± SD	Diff	Students M ± SD	Physicians M ± SD	Diff	Diff
**Internal medicine** ***n***_***s***_ = 44 ***n***_***p***_ = 20	3.83 ± 0.41	3.76 ± 0.59	0.07	3.55 ± 0.65	3.38 ± 0.72	0.17	3.51 ± 0.51	3.43 ± 0.70	0.08	-0.08
**General medicine** ***n***_***s***_ = 24 ***n***_***p***_ = 11	3.68 ± 0.74	3.32 ± 0.28	0.36	3.46 ± 0.86	3.05 ± 0.47	0.41	3.33 ± 0.73	2.93 ± 0.55	0.40	0.17
**Paediatrics** ***n***_***s***_ = 21 ***n***_***p***_ = 7	3.90 ± 0.56	3.37 ± 0.38	0.53	3.74 ± 0.87	3.36 ± 0.85	0.38	3.68 ± 0.61	3.27 ± 0.57	0.41	0.05
**Anaesthesiology** ***n***_***s***_ = 18 ***n***_***p***_ = 11	3.79 ± 0.44	3.63 ± 0.98	0.16	3.75 ± 0.58	4.09 ± 1.04	-0.34	3.83 ± 0.50	3.91 ± 0.90	-0.08	-0.14
**Gynaecology** ***n***_***s***_ = 17 ***n***_***p***_ = 7	3.95 ± 0.38	3.60 ± 0.84	0.35	3.88 ± 0.74	3.50 ± 0.91	0.38	3.45 ± 0.49	3.38 ± 0.97	0.07	0.06
**Surgery** ***n***_***s***_ = 15 ***n***_***p***_ = 14	3.98 ± 0.46	3.65 ± 0.57	0.33	4.18 ± 0.46	4.43 ± 0.55	-0.25	3.91 ± 0.52	3.64 ± 0.46	0.27	-0.07
**Orthopaedics** ***n***_***s***_ = 8 ***n***_***p***_ = 11	4.00 ± 0.60	3.56 ± 0.44	0.44	3.81 ± 0.88	3.86 ± 0.67	-0.05	3.88 ± 0.53	3.28 ± 0.74	0.60	-0.01
**Dermatology** ***n***_***s***_ = 7 ***n***_***p***_ = 10	3.56 ± 0.30	3.83 ± 0.45	-0.27	3.21 ± 0.76	4.30 ± 0.48	-1.09	3.56 ± 0.30	3.75 ± 0.42	-0.19	-0.35
**Radiology** ***n***_***s***_ = 7 ***n***_***p***_ = 9	4.33 ± 0.42	4.50 ± 0.53	-0.17	3.93 ± 0.79	3.56 ± 0.77	0.37	4.06 ± 0.40	4.06 ± 0.50	0.00	0.16
**Mean**	3.87	3.69	0.22	3.70	3.73	-0.02	3.60	3.51	0.17	-0.05

*Diff* Difference

**Fig. 1:**
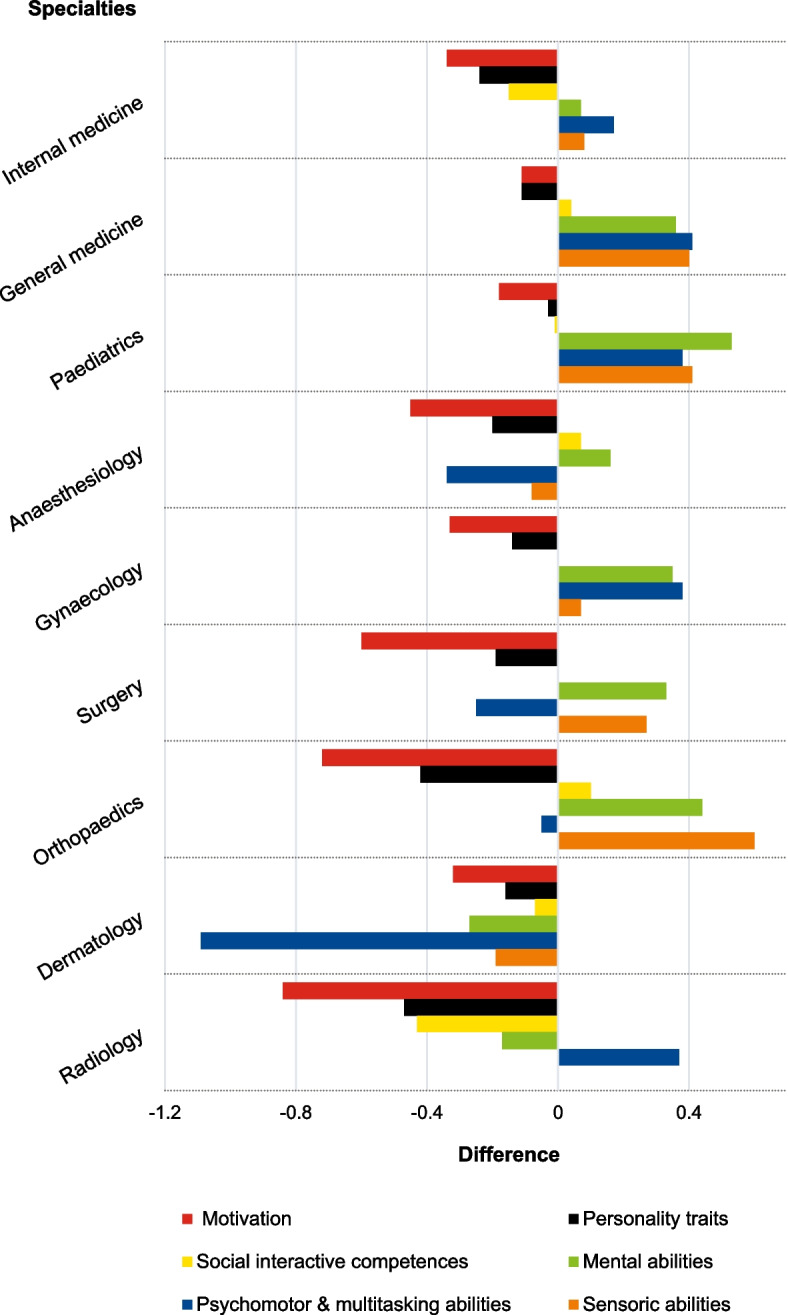
Mean difference scores between students and physicians across specialties

**Fig. 2:**
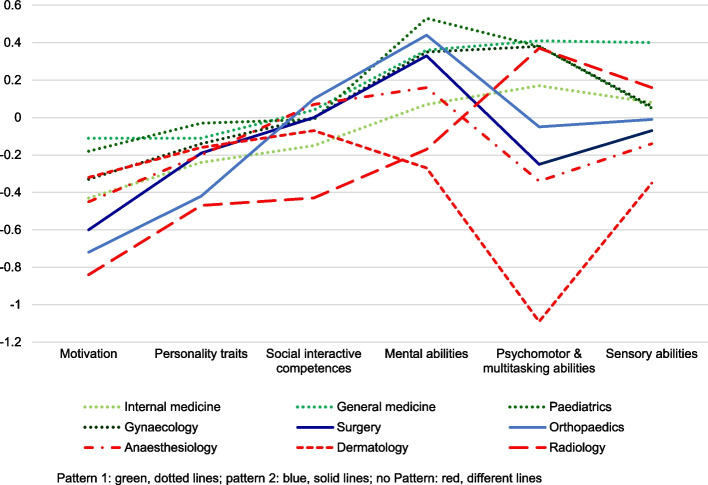
Display of difference score patterns across competence areas and specialties

All difference scores are displayed in Fig. 1. Difference scores for “Motivation” and “Personality traits” were negative (meaning lower scores in the student group) across all specialties ranging from -0.11 (general medicine) to -0.84 (radiology) with an average difference score of -0.44 and from -0.03 (paediatrics) to -0.47 (radiology) with an average of -0.22, respectively. Additionally, internal and general medicine as well as paediatrics and gynaecology all show a similar pattern (referred to as pattern 1) with difference scores for “Social interactive competences” being either negative (internal medicine) or close to zero (general medicine, paediatrics and gynaecology) and difference scores for the remaining areas (“Mental abilities”, “Psychomotor & multitasking abilities” and “Sensory abilities”) being positive (meaning higher scores in the student group). Surgery and orthopaedics only deviate slightly from this pattern with difference scores for “Psychomotor & multitasking abilities” being negative in the student group (pattern 2). All patterns are visualized in Fig. 2.

Anaesthesiology, dermatology, and radiology all show different patterns. Anaesthesiology, while sharing some pattern aspects with the other specialties (“Social interactive competences” close to zero and a positive score for “Mental abilities” like pattern 1 as well as a negative difference score for “Psychomotor & multitasking abilities” like pattern 2), deviates from both patterns with a negative difference score for “Sensory abilities”, making it one of two specialties with that distinction (dermatology being the other). Dermatology deviates from all previously mentioned patterns with negative difference scores for “Mental abilities”, “Psychomotor & multitasking abilities”, and “Sensory abilities”. While both anaesthesiology and dermatology show difference scores close to zero for “Social interactive competences” which is related to pattern 1, thus similar to all other specialties, the difference score for radiology with -0.43 is not close to zero. Furthermore, radiology is one of two specialties (next to dermatology) with a negative difference score for “Mental abilities” while all other specialties show positive scores.

The one-way MANOVA showed a statistically significant difference between the specialties on the combined competence areas, *F*(8, 151) = 2.67, *p* < 0.001, partial *η*^*2*^ = 0.12. Post-hoc univariate ANOVAs showed statistically significant differences between the specialties for competence areas “Mental abilities”, “Psychomotor & multitasking abilities”, and “Sensory abilities”. Post-hoc comparisons with Tukey HSD tests revealed significant differences only for “Mental abilities” and “Psychomotor & multitasking abilities”, but not for “Sensory abilities”. For “Mental abilities”, differences between pediatrics and dermatology (*p* = 0.011), general medicine (*p* = 0.003) as well as internal medicine (*p* = 0.043) and radiology (*p* = 0.043) were significant with difference scores for pediatrics tending towards higher positive values (higher scores for students). For “Psychomotor & multitasking abilities”, differences between dermatology and other specialties except anaesthesiology, orthopaedics, and surgery were significant with higher negative difference scores (lower scores for students) for dermatology.

**Fig. 3:**
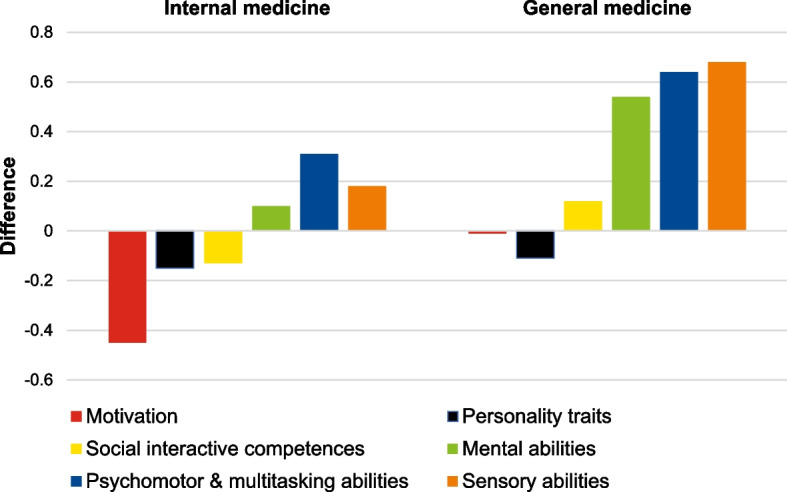
Mean difference scores per competence area for internal medicine and general medicine between physicians and all students independent of their specialty choice

All 197 participants combined showed the following competence expressions for the six competence areas: “Motivation” = 3.91 ± 0.54, “Personality traits” = 3.74 ± 0.36, “Social interactive abilities = 3.78 ± 0.35, “Mental abilities” = 3.86 ± 0.52, “Psychomotor & multitasking abilities” = 3.69 ± 0.75, and “Sensory abilities” = 3.61 ± 0.58. Computing mean difference scores across competence areas for specialties independent of students’ choices revealed the lowest difference score for internal medicine (-0.02). The difference score for general medicine (0.31), was the most positive with a higher student score than physician requirement score. Difference scores in all competence areas of the two specialties are shown in Fig. 3.

## Discussion

The goal of this study was to determine student-specialty fit and compare different medical specialties regarding the overlap of students’ self-assessment and physicians’ requirements of different competence areas for specialty training. For all specialties, physicians’ requirements regarding “Motivation” and “Personality traits” were higher than the respective students’ self-assessments. These high expectations of practicing physicians are in line with developments regarding the medical profession where non-technical or knowledge based qualities and skills are becoming increasingly important for good clinical practice [35, 36]. Students recognized factors like enthusiasm and commitment to a specialty as relevant [37] and rated competences other than clinical knowledge and skills as important for the medical profession [38]. In the present study, students’ “Motivation” scores were consistently lower than physicians’ requirements in that area, but still highest among all competence areas compared to the other student scores which emphasizes students’ motivation for their respective specialty of choice. Even though students are aware that non-technical qualities and skills are important in the medical profession across specialties, undergraduate medical curricula should further support the development of these skills to support students to reach a level required in the transition to postgraduate training [39].

Expression of “Social interactive competences” was rated higher by students than rated by physicians from most specialties as requirement, or differences between students’ and physicians’ ratings were close to zero. Students seem to have sufficiently developed social competences as another non-technical skill by the end of their undergraduate training. Developing and improving interpersonal communication skills is widely implemented in undergraduate medical curricula [40]. Furthermore, “Social interactive competences” are regarded as basic competences by undergraduate medical students, especially the skill to structure information in communication [20]. In our study, requirements for “Social interactive competences” are met for most specialties at the end of undergraduate training except for radiology, internal medicine, and dermatology. In these specialties, requirements for “Social interactive competences” are rated higher by physicians than the personal competence level assessed by students who wish to choose these specialties for postgraduate training. For radiology residents, for example, courses are offered in their postgraduate training to improve their oral presentation skills [41, 42].

The three competence areas “Mental abilities”, “Psychomotor & multitasking abilities”, and “Sensory abilities” showed lower physician scores than student scores for internal medicine, general medicine, paediatrics, and gynaecology (pattern 1). This indicates that students near graduation fulfil or even exceed the requirements in these more technical and knowledge-based competence areas when choosing one of these specialties. Our findings for all pattern 1 specialties match their categorizing as person-oriented whereas the other specialties included in this study can be regarded as more technique-oriented [43, 44]. The results thus show that students’ scores are sufficient for technical and knowledge-based competences at the end of undergraduate training when a person-oriented specialty is chosen. Physicians of most technique-oriented specialties expect higher scores in at least one of the technical competence areas. Surgical skills (in orthopaedics and surgery) as well as specific psychomotor skill applications and monitoring processes (in dermatology and anaesthesiology) could attribute to higher score expectations in “Psychomotor & multitasking abilities”. Higher score requirements for “Sensory abilities” in anaesthesiology possibly relate to specific auditory and visual cue perception during monitoring [45]. Radiologists’ requirements for “Mental abilities” are potentially exceeding students’ assessments due to the extensive use of imagery in the field which is practiced in postgraduate education [46].

Regardless of students’ specialty choices, we found the lowest difference score for internal medicine (-0.02) and the difference score for general medicine (0.31) was the highest in favour of student scores. This implicates that overall undergraduate training seems to provide students with good person-oriented skills while specific skills for technique-oriented specialties are less covered or have to be acquired during postgraduate training in such specialties [47]. This is also evident in the variance of physicians’ scores in different specialties while students’ scores varied less. Differences identified in the fitting process of students to different specialties also provide evidence that the acquisition of some specialty specific skills and qualities occurs during residency training and medical practice [48, 49]. Adapting the undergraduate curriculum towards enhancement of non-technical qualities for all students while offering more differentiated contents regarding specific skills-based competences for students with an interest in more technique-oriented specialties could provide better students’ fit with their specialty of choice.

With a total of 197 student assessments sufficient data was available for analysis of nine specialties, allowing comparisons between specialties, which is a strength of this study. Only for the competence area “Personality traits” difference scores increased with age, representing the effect of personality maturation [50]. Contrasting different specialties is an important step when determining student-specialty fit. Computing difference scores allows comparisons between the specialties with an underlying metric, expressing the fit between students and physicians. However, although both student and physician scores are on a 5-point Likert-scale, self-assessment of competences and requirement assessment of competences differ in their respective perspectives. While students were asked to assess their own competences compared to other undergraduate students, physicians were asked to rate the statements according to their relevance in their respective field. Therefore, the difference scores are potentially biased by methodological aspects, which is a limitation of our study. Moreover, self-assessment is generally biased and does not necessarily display the actual competence [51, 52]. Higher or lower student scores can thus also be attributed to inaccurate assessments. Also limiting the interpretation of results, difference scores resulted from subtracting physician scores from student scores and were thus not displayed as absolute values. Since negative and positive scores were included, mean difference scores around zero can indicate both low difference scores overall or high difference scores for different competence areas in both directions also resulting in mean scores close to zero. However, we chose this representation as absolute difference scores would have limited the interpretation of good and bad fit since students’ exceeding physicians’ requirements would not have been possible. Some physician scores are higher than others despite having similar requirements (e.g. technique-oriented specialties), inducing a bias towards higher difference scores for these specialties. It remains unclear what influences these high scores since no qualitative assessment of potential influence factors was included in the study. This limits the interpretation of results since high difference scores are potentially biased. Furthermore, it encourages future research to assess differences between specialties that might account for these high score differences that are yet unclear.

Despite these limitations, students’ matching of their competences with the required competences for their specialty of choice can provide them with the opportunity to customise their learning with respect to specialty specific requirements. Self-assessment of competences cannot replace medical educators’ assessment of students’ competences. However, when items are well chosen students’ self-assessment can become more realistic [53]. Therefore, self-assessment with R-Track could be used longitudinally, e.g., starting in year four of undergraduate training, by students to identify their current competence profile and match it with required profiles of specialties of their interest. If, for example, a student who wishes to eventually choose surgery for residency training, notices in year four, that he or she is lacking competences in the area of “Psychomotor & multitasking abilities”, the student could plan his or her further studies with a focus to improve facets of competence from this area, e.g. in electives. On the other hand, if a student worked to reach the required competence profile for surgery but ends up with a dermatology match, he or she will be able to identify competence areas with the R-Track where his or her individual profile does not match dermatology requirements and he or she can focus on improving competences needed in specific areas during postgraduate training. As self-assessment is an important feature of life-long learning in medicine, the R-Track can provide guidance for undergraduate students to identify competence areas for improvement to reach a good competence match with the competence profile of a specialty they wish to choose for residency training. If residents have to work in a specialty for which their R-Track profile does not match the required profile of the respective specialty, they can easily identify the competence areas they need to focus on during postgraduate training to reach a better match for their specialty.

## Conclusions

Comparing students’ competence profiles with the required competence profiles of specialties students are interested in for residency training can provide students with new insights with respect to their quality of fit. Overall, students show better fit with specialties that are person-oriented. “Motivation” and “Personality traits” are important competence areas for all specialties and seem to need a more prominent focus in undergraduate training. “Mental abilities”, “Psychomotor & multitasking abilities”, and “Sensory abilities” show difference scores in favour of physicians’ requirements for more technique-oriented specialties. This highlights the focus on basic skill development in undergraduate training for students who are interested in such specialties or provides evidence that some specific skills will be developed during residency. Future studies should aim to assess competencies needed for a good specialty fit from both students and educators involved in undergraduate and residency programs.

### Supplementary Information

**Additional file 1:** Competence areas and items of R-Track.

## Data Availability

All data and materials are available from the manuscript.

## Abbreviations

ANOVA: Analysis of variance
MANOVA: Multivariate analysis of variance
R-Track questionnaire: Requirement-Tracking questionnaire
Tukey HSD test: Tukey honestly significant difference test
