# Supplementary material for: Matching of advanced undergraduate medical students’ competence profiles with the required competence profiles of their specialty of choice for postgraduate training
Source: BMC Med Educ. 2023 Sep 7;23:647. doi: 10.1186/s12909-023-04632-3 (PMC10485971; doi:10.1186/s12909-023-04632-3)
Supplement: Supplementary file 1 — Additional file 1: Competence areas and items of R-Track. [file 12909_2023_4632_MOESM1_ESM.docx]

**Supplement 1: Competence areas and items of R-Track**

| **Competence area** | **Items** |
| --- | --- |
| Motivation | Achievement motivation  Endurance  Expertise  Reliability & discipline  Thoroughness |
| Personality traits | Cooperation / Agreeableness  Creativity  Emotional stability  Flexibility  Honesty  Independence and autonomy  Modesty  Openness to novelty  Openness to other people / cultures  Prudence  Risk orientation  Tolerance to frustration |
| Social interactive competences | Coaching and Mentoring  Conflict management  Coordination & decision making  Delegation / Delegating  Diplomacy  In need of harmony  Manners & common decency  Norms and values orientation  Orientation towards patients  Persuasiveness  Presentation  Resistance to monotony  Sanctioning  Self-confidence  Sense of humour  Sociability  Sovereignty  Stress resistance  Structuring information  Tactfulness  Willingness to help |
| Mental abilities | Clarity of speech  Concentration  Facility for languages  Logical reasoning  Mathematical reasoning  Memory capacity  Numeracy  Problem comprehension  Reading comprehension  Spatial orientation  Spatial visualization  Verbal expression  Verbal understanding  Written expression |
| Psychomotor & multitasking abilities | Multitasking capacity  Psychomotor coordination |
| Sensory abilities | Auditory discrimination  Comprehension  Hearing sensitivity  Near vision  Perceptual range  Perceptual speed  Range of field vision  Selective attention  Visual imagination |
